# ‘Every day I worry about something’: A qualitative exploration of children’s experiences of stress and coping

**DOI:** 10.1111/bjhp.12387

**Published:** 2019-08-26

**Authors:** Tara J. Cheetham‐Blake, Hannah E. Family, Julie M. Turner‐Cobb

**Affiliations:** ^1^ Department of Psychology University of Bath UK; ^2^ Department of Pharmacy and Pharmacology University of Bath UK; ^3^ Present address: Faculty of Health Sciences University of Southampton UK; ^4^ Present address: Department of Psychology Bournemouth University UK

**Keywords:** child, coping, parent, resilience, stress, qualitative

## Abstract

**Objectives:**

Most research investigating children’s experiences of stress and coping has utilized a quantitative approach. This study aimed to examine children’s experiences of stress by conducting interviews with children and their parents.

**Design:**

Dyadic child–parent interviews, embedded within a multiphase design.

**Methods:**

Thirty‐eight children (22 boys) aged 7–11 years and 38 parents (34 mothers) completed in‐depth dyadic interviews about stressful life events, adversity, and coping, analysed using inductive thematic analysis with a phenomenological lens.

**Results:**

Four themes emerged: (1) navigating the social minefield; (2) pressure to thrive in the modern world; (3) fear of the unknown; and (4) learning life’s lessons. The first suggested that social relationships are a major feature of children’s stress experiences; however, social support was also found to be a beneficial coping mechanism. The second theme highlighted multiple sources of pressure on young children (including school, extracurricular activities, pressure from self and others); the impact of such pressure was dependent upon children’s coping resources. The third theme emphasized the difficulty of coping with novel stressors, and how awareness can help reduce this fear. The final theme highlighted important lessons that children can learn from stressful experiences and how to cope with stress.

**Conclusions:**

This study addresses the importance of the person and context‐dependent nature of stress and coping in order for children to survive and thrive following stressful experiences. These findings contribute to existing knowledge that could be used to develop a toolkit for coping with stress, designed specifically for children, parents, schools, and services.

**Statement of contribution**

***What is already known on this subject?***

Stress experienced in childhood can have a significant impact on psychological and physiological outcomes across the life course. It is known that individual differences are vital for understanding the effects of stress on health, for children as well as adults. Qualitative methods enable deeper understanding of children’s experiences of stress and coping.

***What does the study add?***

- Depth and breadth to understanding children’s experiences of stressful events.
- An individual differences focus on the early stress experience that is frequently overlooked.
- Support for the use of a dyadic interview approach for assessing children’s stress experiences.

Stress experienced in childhood can have a significant impact on psychological and physiological outcomes across the life course (Braveman & Barclay, 2009; Nurius, Green, Logan‐Greene, & Borja, 2015). Childhood stressors can take the form of major life events, such as parental divorce or moving house, through to daily hassles, including falling out with friends and struggling with schoolwork. These stressful early experiences can have a negative effect or may result in the development of positive coping strategies, depending on situational and individual factors, which can act as psychosocial moderators between stress and health (Turner‐Cobb & Steptoe, 1998) with implications for later life. Coping strategies are known to evolve across the life course, with different strategies employed in childhood, adolescence, and adulthood (Aldwin, 2009). Therefore, it is imperative to explore children’s early experiences of stress and coping.

Much of the literature examining childhood stress and coping utilizes quantitative methods, including physiological responses to laboratory stressors and life event scales. While questionnaire‐assessed life events report the number of major stressors children have encountered, they do not reveal the extent to which the stressors have influenced the child, capturing data on only certain aspects of children’s stress experience (Holmes & Rahe, 1967). The relatively small amount of qualitative research that has been conducted on stress in childhood has focused on the effect of different types of stress (e.g., chronic vs. episodic) on psychiatric disorders (Gershon *et al.*, 2011), the impact of illness‐related stress on depression and anxiety (LeBovidge, Lavigne, & Miller, 2005), and comparison of quantitative (life events checklist) and qualitative measures (life events interview) (Wagner, Abela, & Brozina, 2006). These studies provide a basic overview of particular aspects of children’s stress experience; research questions focus on mental health or methodology and they fail to explore the depth or breadth of children’s stress. Research that goes beyond describing the nature of stressors and investigates how children make sense of stressful experiences is needed to advance the field of child stress research.

The importance of interviewing child–parent dyads together has been highlighted as providing the most illuminating and rich data as children and their parents interact to build a narrative (Cheetham & Turner‐Cobb, 2016). For example, children often struggle to remember examples of times when they felt stressed but parents are able to prompt memory for specific events. Once recalled, children are often very good at describing how they felt about an event, something the parent would have been less able to do. This ‘story scaffolding’ generates a rich depth of data which would not be possible to access if either of the dyad were not present (Irwin & Johnson, 2005).

The present study aimed to better understand children’s experiences of stress, adversity, and coping, through in‐depth interviews with child and parent dyads to ensure that the perspectives of both children and parents could be garnered.

## Method

### Design

This study is part of an embedded multiphase design encompassing qualitative and quantitative data collection, in which children completed questionnaires and interviews about their experiences of stress and coping. Interview data are reported in this paper; questionnaire data are reported elsewhere (Cheetham‐Blake, Turner‐Cobb, Family, & Turner, 2019).

### Participants

Ethical approval for this study was granted by the University’s Psychology Department ethics committee. An opt‐in recruitment method with press releases in local newspapers, advertisements in schools, on relevant websites, and through social media platforms was used to recruit healthy children and their parents.

Thirty‐eight children (16 females, aged 7–11 years), each accompanied by one parent, took part in the interviews. Thirty‐four of the participants were interviewed with their mothers, and four were interviewed with their fathers. The sample was White European with a median socio‐economic status (SES) score of 54 (possible range = 8–66; higher scores correspond to higher SES) and IQR of 11 (Hollingshead, 1975). Table 1 shows age and sex of the sample.

**Table 1:** Participant numbers split by demographics (age and sex) of the study sample

	Males (22 participants)	Females (16 participants)
7 years	C12, C16, C32, C34	C14, C22, C33
8 years	C17, C26, C30	C2, C7, C20, C27, C29
9 years	C19, C31, C37	C5, C15, C24, C36, C38
10 years	C9, C10, C11, C21, C25	C23
11 years	C3, C4, C6, C8, C13, C28, C35	C1, C18

Note

C3, C8, C24, and C25 were interviewed with their fathers; the other 34 children were interviewed with their mothers.

### Measures

#### Questionnaires

Socio‐demographic data (child age, sex, ethnicity, and parental SES) and details of children’s experiences of stressful life events (Social Readjustment Rating Scale; Holmes & Rahe, 1967), daily hassles (Children's Hassles Scale; Kanner, Feldman, Weinberger, & Ford 1987), and coping strategies (Kidcope; Spirito *et al.*, 1988) were collected. Children completed questionnaires with the assistance of their parents. Questionnaire topics were used to guide the interview protocol.

#### Interview protocol

Several interview protocols investigate early life stress and adversity, health, and coping strategies in young children (Gershon *et al.*, 2011; LeBovidge *et al.*, 2005; Wagner *et al.*, 2006). However, none of the existing protocols fully cover all topics together in one interview schedule and they focus on the impact of stress on mental health. Existing protocols also often use parents as proxy respondents for their children. It is important to speak directly to children about their experiences, rather than relying on parental proxies (Docherty & Sandelowski, 1999). In the few instances when children were interviewed in previous literature, they participated separately from their parents (LeBovidge *et al.*, 2005).

To address these issues, a new dyadic interview schedule was developed for the present study. This enabled the interviews to be semi‐structured, with some predetermined questions and topics, but also allowing the interviewer’s questions to be led by topics that participants felt to be significant or relevant. The interview questions complemented the topics covered in the questionnaires, but were not constrained by them; the questionnaire was a starting point for a broader discussion. For example, the first part of the interview focussed on stressful experiences, so the researcher would ask about a life event or daily hassle that the participant had indicated in the questionnaire to have been highly stressful. Table 2 shows interview questions and prompts.

**Table 2:** Interview protocol detailing the questions and prompts for each of the five topics covered in the interviews

Topic	Questions
1. About you (rapport building)	Could you tell me a bit about yourself and your family? Prompts: Who do you live with? Has this changed since you were little? What kind of things to do like doing at the weekend? What subjects do you like at school? What are your hobbies/things you like doing? *Complete ‘about you’ scale*
2. Stressful life events and daily hassles	For stressful life events: You mentioned that *(type of event)* happened. Could you describe what happened? How did you/your family feel about *(the event)*? Prompts: Length of the event? Coping: Could you describe how you coped with *(the event*)? For example, what did you do to make yourself feel better? How do you think *(your child)* coped with *(the event)*? Comparisons: Could you compare what you felt like before *(the event)* and how you feel now? For example, compare how you felt about school/relationships with friends/family before and after. Could you compare your feelings/behaviour before and after *(the event)*? Effect on present life: Could you compare how you/your child deals with new challenges or problems before and after *(the event)*? Could you describe how you/your child reacts if they are reminded of *(the event)*? Prompts: Interference with day‐to‐day life? For daily hassles: You said that *(type of hassle)* made you feel *(quite bad/very bad)*. Could you tell me a bit more about it/how it made you feel? Prompts: Are there other times when you’ve felt similar to that? Are there any other stressful events that have happened to you that we’ve not talked about? Prompts: How old were you/your child when this happened?
3. Coping	When you told me about how you coped with that problem on the coping questionnaire you said that you found *(top three strategies)* most helpful. Go through each strategy in turn: Could you tell me about any other times you’ve used this strategy? Can you tell me how you normally cope with problems? What do you normally do if something bad happens to you? Prompts: Can you tell me a bit more about that? Do you use any of the things on the list? Can you describe a time when you’ve used this strategy? Can you describe how you feel once you’ve used them?
4. Health and illness	Can you tell me a bit about what it’s like to be ill? On the health questionnaire you said that you/your child *(go through each question, e.g., had some health problems)*. Could you tell me a bit more about that? *For each specific illness: Complete health and illness scale*
5. Early life events (questions for the parent) Note: the child was asked the closing questions and left the interview prior to these parental questions)	Were there any stressful events that happened during your pregnancy? Were there any stressful life events that happened to you or *(your child)* during the first year of their life? Prompts: How did these events make you feel?
Closing questions	Is there anything I’ve missed or not asked you about that you think is very important? Do you have any final questions or comments?

Adaptations were made to the interview format and style to make it more appropriate for young children and reduce fatigue. In addition to the use of brief scales to break up the interview, the protocol comprised a combination of open and closed questions, with prompt cards for the interview topics placed in front of the participant throughout. These techniques are recommended as best practice for dividing up interviews with young children into manageable sections and keeping children engaged and focused throughout (London School of Economics, 2010; Shaw, Brady, & Davey, 2011).

### Procedure

Parents were given detailed information about the project during their initial contact with the researcher; the interview was scheduled at least a week after the initial contact so that parents, and children had adequate time to consider their participation. The researcher met with participants at their homes or in a meeting room at the University. Parents received an information sheet to read while the researcher verbally explained the study to the child. Verbal assent was gained from the child and written consent from the parent. The child–parent dyads completed the questionnaires together and then took part in the interview that ranged in length from 20 to 55 min depending on how much life stress was reported and their ability to expand on answers; the average length of the interviews was 30‐min. Interviews were audio‐recorded and transcribed verbatim. Afterwards, children and their parents were verbally debriefed and given a £10 shopping voucher to thank them for their participation.

### Data analysis

Interviews were analysed in NVivo version 10: QSR International. An inductive thematic analysis was conducted using a phenomenological lens (Guest, MacQueen, & Namey, 2012). This approach was selected due to the exploratory nature of the study, as no interviews previously addressed all the topics. This exploratory nature is part of the underlying philosophical presuppositions of the phenomenological approach; its purpose in the present study was to describe children’s lived experiences of the phenomenon of stress and coping (Creswell, 2013). The researchers did not want to impose a predetermined framework but allow the data to determine the analysis structure and to understand the sense that children made of their experiences of stress and coping. Analysis involved the lead researcher familiarizing themselves with the data (in part through interview transcription), generating initial codes, searching for themes, reviewing themes, defining and naming themes, and writing up the analysis (Braun & Clarke, 2006). Transcripts were fully coded and a coding manual developed and used to guide the analysis.

### Inter‐rater reliability

Two independent qualitative researchers coded six of the transcripts (15% of the data) chosen at random. The lead researcher met with each coder to compare all the codes created. Any codes that appeared in one person’s analysis but not the others were discussed, and a decision made about their inclusion. This method of code checking (Silverman, 2013) yielded a high level of congruence in the codes developed by the coders and lead researcher; all original codes were agreed upon, the wording of five codes was improved upon, and five new codes were included in the analysis. For further details of how the study complied with quality criteria please, see the Appendix which maps the study to the COREQ criteria of reporting qualitative research.

## Results

**Figure 1:**
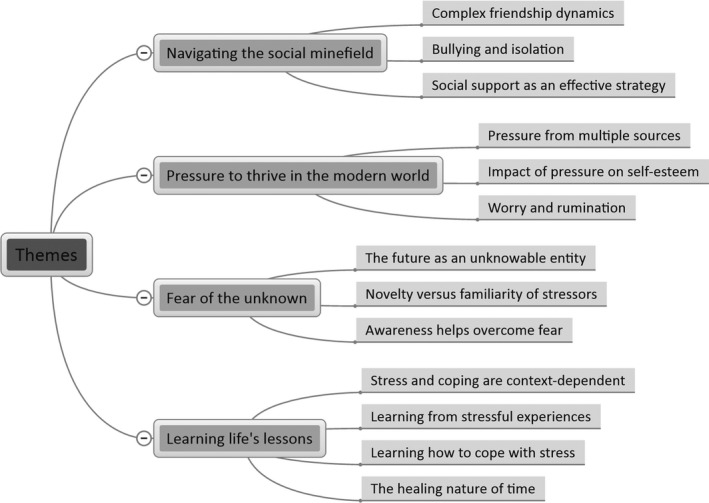
Visual representation of the overarching themes and associated candidate themes from the thematic analysis of the data.

Four overarching themes emerged from the data: (1) navigating the social minefield; (2) pressure to thrive in the modern world; (3) fear of the unknown; and (4) learning life’s lessons. The first two themes represent the broader social elements of children’s stress experiences, whereas the latter two themes reflect more individual features of children’s experiences. Each theme encapsulates two to four candidate themes (see Figure 1).

All quotations are verbatim and in order to maintain anonymity, identified by a participant number alongside ‘C’ for child participants and ‘P’ for parents. The participant identification number for each parent corresponds to their child’s identification number, for example, P1 is the parent of C1 and so on.

### 1. Navigating the social minefield

For many children in this study, their social world was a difficult area of their lives to navigate. Managing dynamic relationships with people was an important skill for children to learn, especially as their relationships with friends were often far from smooth. Bullying was also a source of stress for children, particularly in relation to the negative feelings that result from bullying, and the potential reasons for its occurrence.

#### Complex friendship dynamics

The dynamic and changing nature of children’s friendships was evident in how they spoke about their friends, for example, ‘sometimes he annoys me but sometimes he’s also my friend’ (C10). Friendship was seen as fluid, with falling out as a normal everyday part of friendship. Despite their frequency, these fallings out were not taken lightly. Children found falling out upsetting, difficult, and stressful with several participants referring to fallings out as ‘break ups’ (C7, C23). In these children’s narratives, friendships were restored after parents or teachers intervened (C22, C29, C30, C35), children talked through their problems (C20), or forgot about the falling out (C38).

Levels of friendship and the social hierarchy were an interesting feature of the interviews. Distinctions were made between ‘best’ friends and regular friends (C23). ‘Enemies’ was a term used by some participants (C26, C28). There appeared to be a need to categorize friends based on closeness in a personal social ranking or hierarchy, with fallings out often occurring when a friend’s actions suggested they did not value their place in the social ranking (C26).

#### Bullying

Many children experienced negative social interactions such as bullying. The bullying described in the interviews ranged from mean comments and disrespectful behaviour, ‘she would also back‐chat you and be horrible about people’ (C1), destruction of personal property, ‘it’s like [participant] had this thing that he thought was cool so they wanted to destroy it’ (P6), and physical fighting and violence (P28). The language children used to describe bullying and its various forms are evidenced in this discussion between child and mother.

P Your use of the term ‘beat me up’ also means speaking badly at you doesn't it (.) it's not only a physical activity (.) if they look at you in a certain way it means ‘beat you up’

C And they like talk about me (.) ‘oh yeah [participant]'s a horrible person don't go and play with him’ (.) just behind my back when I'm not there (C28 and P28).

Bullying often had very serious consequences, with some children being so worried that they could not eat or sleep (C1), feigning illness or running away from school to avoid the bullies, and even changing schools (C2, C6, C28). Often bullying led to feelings of exclusion and isolation (C6, C12, C23, C28, C31, C35).

Participants frequently discussed why they thought they were bullied, often because they were ‘different’ from other people (C28) or because the bullies were trying to hide their own insecurities (C36). One participant felt his bullying was caused by his perceived social standing and that when he moved schools he felt a change in his social status, ‘a definite elevation, I’m not sure how to describe it but I was respected more. I was treated like a person’ (C6).

### 2. Pressure to thrive in the modern world

Children experienced pressure to do well at school, in extracurricular activities and taking responsibility for their own health; placed on them by themselves (internal pressure) and by those around them (external pressure). This pressure to thrive was shown to have positive effects (e.g., increased motivation and self‐worth) and negative effects (e.g., negative self‐esteem, worry, and rumination) on children’s psychological well‐being.

#### Pressure from multiple sources

School‐related pressure was a major feature of children’s narratives, especially in relation to struggling with schoolwork in the classroom (C15, C17, C23, C31) and difficult homework (C21, C25, C29, C34). Examinations and revision were reported to be a particularly stressful aspect of school (C4, C21, C36).

Ever since year five almost the school’s been preparing us for an exam to go to the same school, just into the senior school and then it all came down to this one day almost. It was a lot of pressure and it was quite stressful because we did lots of mock exams. (C36)

This participant’s stress resulted from combined internal and external pressure, wanting to do well for himself but also feeling pressure from the school. Parental pressure also played a role in children’s narratives, for example, participating in a sporting event that they did not want to take part in (C15).

Sources of pressure external to schoolwork included rehearsing for a play (C1), competing in gymnastics competitions (C16), public speaking as part of a role on the school council (C30), and health‐related responsibility (C7). As children’s perceived competence increased, they were given more responsibility for their own health, for example, in relation to food allergies (C7, C10) and avoiding situations which would exacerbate eczema by ‘trying to make the right choices as to whether you should go on the field to play with your friends or just stay on the playground’ (P5).

#### Impact of pressure on self‐esteem

The pressure to thrive could have a positive or negative effect on children’s self‐esteem and feelings of self‐worth, depending on the coping strategies available to them. Many of the children were motivated to overcome adversity to demonstrate their self‐worth to others and themselves, for example, one child went above and beyond when applying for an IT support role at school by preparing a presentation to show to her teachers (C27). In response to not doing well during a swimming lesson, one participant was primarily concerned about what others would think of their ability but soon positively reframed the situation in terms of future opportunities to showcase their skills:

I didn’t have a good day, so I told myself not to worry because there’s always another chance to prove that you’re better than that particular Saturday. (C14)

Pressure to achieve can have a negative impact on children’s well‐being and self‐esteem. Some parents felt that the modern school system put too much pressure on young children and turned to alternative forms of schooling (such as forest schools) which were noted for their ability to improve children’s self‐esteem, feelings of self‐worth, coping strategies, and behaviour (P26, P28, P29) and provide a way for parents to introduce some balance into children’s lives.

P He was off school for one day a week to learn in a different way, in a different environment, and that helped with his emotional development and his ability to cope with stress and I think that has given him a lot of resilience, and he now knows he can cope with anything

C I try to have positive thoughts and that helps (C13 and P13).

#### Worry and rumination

One of the negative outcomes of this pressure to thrive was worry and rumination, a key feature in the narratives of the children in this sample. Some participants remarked that they worried about everything (C31), and that worrying was a part of their daily life, ‘every day I worry about something: school stuff, home stuff, everything’ (C22). These worries and anxieties often transformed into rumination, with children dwelling on particular stressors. Parents commented on how their children ruminated on problems for a long time (P25) and one remarked that their child ‘carries stuff with him’ (P37). One of the features of rumination noted by children in the sample was how well they coped with things during the day but ruminated at night (C18, C31).

When I go to sleep I panic, like as soon as my head hits the pillow if I don’t have internet and I can’t go on YouTube, my head, I start on like a mini panic attack cos you think about everything and it’s really annoying. You need to make your life perfect but it’s never going to be perfect, but you really start panicking and it’s not very nice (C18)

As shown here, rumination clearly has a negative impact on the participant’s sleep and well‐being. Although she does describe a distraction coping mechanism, her concerns about being good at everything and wanting to be perfect are an indication of the pressure that she feels. The type of distraction chosen by the participant (the internet and YouTube videos) could also be increasing the pressure to be perfect, as social media often perpetuates a positive bias with contributors only presenting highlights of their life.

### 3. Fear of the unknown

Unknown stressors were a significant feature of children’s narratives about their experiences of stress, particularly in relation to coping. If a stressor was familiar, it was deemed as easier to cope with whereas a novel stressor was harder to adapt to. A strategy that was found to be helpful for dealing with unknown future stressors was increasing awareness through information gathering.

#### The future as an unknowable entity

The unknowable and unpredictable nature of the future can lead to feelings of worry. Children are still learning about stress and how to cope with it and therefore have fewer experiences to draw on to make estimations about the future. Children reported worrying about major life events, such as changing schools (C13, C26), a parent being away for long periods of time (C7, C34), and parents changing their jobs (C30, C31). Smaller hassles that affected children’s daily routines were also found to be stressful, for example, when a participant was summoned to the head teacher’s office but did not know why. In this extract, it is evident that it was the unknown nature of the situation that made the experience more stressful.

I went in and I was quite scared cos I didn’t know what I was going to talk to her about, and I thought it was a different situation that I was going to talk to, but it was a situation I wasn’t actually ready for (C20)

#### Novelty versus familiarity of stressors

Experiencing an event for the first time can feel more challenging than an event that is familiar, and this link between stress and novelty was referred to by several of the participants.

I felt really scared and nervous [about giving a speech] cos it was my first time standing in front of the class. Other people like my friend, it's about like her fourth time and it was my first (C30)

Numerous participants remarked on stressful situations which they were familiar with, such as people making mean comments to them: ‘cos it happens so frequently I don’t really feel that offended’ (C8) and in response to a recurring illness ‘I can cope because I had it a lot’ (C15). Familiarity with an event, even an unpleasant one such as bullying or illness, made participants better able to cope with it. Therefore, experiencing stress can sometimes have beneficial outcomes in terms of reducing novelty and improving coping strategies.

#### Awareness helps overcome fear

One method for making novel or unknown situations more bearable for children was through increasing awareness and knowledge by gathering information about the stressor. One participant described, with great insight, the benefit of awareness for her ability to cope with her father’s terminal illness and death.

I already knew that he had an illness though so that kind of helped, but I know a few people who have had it happen sudden…they don’t like to talk about it cos, he literally just died, their dad died just suddenly, they didn’t have any warning (C1)

This participant noted the benefit of knowing that the death was going to happen and being able to mentally prepare for it, comparing her advance knowledge favourably to the experience of someone whose father died unexpectedly. This participant’s ability to positively reframe an immensely stressful experience, and even point to others for whom the situation was worse, highlights how well she has coped and the resilience to adversity she displayed.

Enhancing awareness through information gathering was found to be a helpful coping mechanism by many of the participants, for example, one participant asked his older sister questions about the new school he was starting so he could prepare for his first day (C16). Increasing awareness using the knowledge and expertise of others was a source of comfort, particularly in relation to novel experiences, such as a school caving trip, ‘they were really prepared. They made us hold on to a rope so we’d know where we were going’ (C1).

### 4. Learning life’s lessons

The context‐dependent nature of stress was evident in children’s narratives with an emphasis placed on how major life events and daily hassles were perceived differently by different people. The ability to learn from stressful experiences and how to cope with stress are two important life lessons that can be acquired during this time. The passage of time was also found to be helpful in children’s recovery from stress.

#### Stress is context‐dependent

The type of stressor was frequently noted when children discussed the context of their stress and coping, for example, whether the stressor was a major life event or a daily hassle. For some participants, major life events were more stressful than daily hassles, ‘he copes very well with the day to day stuff but if there’s a big change coming he gets anxious about that’ (P32). Conversely, some children were fully capable of dealing with major life stress but did not cope well with daily hassles and stressors (P2, C11, C18, C36): ‘I’d say that some of the day to day roller‐coasters of emotions can be quite stressful’ (P20). Similar comments were made by other parents who had noticed that their children struggled most with daily hassles, such as falling out with siblings which ‘has the biggest continued impact on her, on an almost daily basis’ (P5, also C5, C7, P17).

#### Learning from stressful experiences

Children experience a wide range of adversities which, although stressful, can be beneficial in terms of learning life skills. For example, one child’s experience of bullying was very upsetting but the parent hoped that it could also be viewed as a learning experience, ‘I like to think that, in the long term, she will learn lots from it’ (P2). In the sample, parents often commented on the importance of children viewing their stressful experiences as life lessons. Examples included a bad gymnastics competition being a way to learn that more practice was needed for the next competition (P20) and that some children may appear overly confident about an exam to hide their own nerves which is a ‘useful life lesson as you learn the filter to put on that stuff’ (P36). Children also used this technique themselves, as one child explained, being told off by a teacher, ‘it feels bad but it also feels ok because it means that if you get told off you know that for next time you don't do that’ (C27).

#### Learning how to cope with stress

Problem‐focussed coping skills such as problem‐solving, cognitive restructuring, and social support were widely used by participants and this was encouraged by parents. Participants sometimes used several coping strategies to deal with a problem, which takes the form of problem‐solving and cognitive restructuring in the example below.

I think he’s more of a try to sort out the problem, try to see why it went wrong so that it doesn’t happen the next time, definitely tries to control himself. He does try and see the good things, you try and see what you can learn from it don’t you? (P16)

Over half of the participants reported using social support as a coping mechanism during times of stress. For several participants (C1, C38), it was not just the act of sharing their problems with another person that they found beneficial, but the process of feeling listened to, understood, and receiving reassurance. Verbal reassurance included assuring children that things would be okay (C38), that when bad things happened ‘it’s not the end of the world’ (C17, also C37), and help with refocussing and restructuring their thoughts in a more positive way (P14, P36). Reassurance was also gained through physical closeness; for some participants, merely being in the presence of their social companions was comforting (C16) and playing with friends helped distract them (C11, C32). Hugging was something that many children found particularly comforting, whether it was hugging their parents, friends, pets, or favourite toys (C15, C17, C20, C26, C31).

Emotion‐focussed coping strategies were also discussed by children and their parents, for example, blaming others, repressing negative thoughts, crying, shouting and screaming, smashing things, fighting, and violence towards oneself.

One more tactic that I sometimes do, I’ll go outside sometimes and like smash a ball, kick the ball as hard as I can or something. If you’re all contained just let it all out and then once I run around and get tired that’s when the problem goes away (C10)

For this participant, releasing anger by kicking an inanimate object was a cathartic experience that helped him to recover from stressful experiences. Another cathartic experience for many participants was crying, allowing them to release their feelings and diminish the impact of the stressor (C35). Creative techniques such as drawing and writing about one’s feelings were also used as a form of emotional expression to aid coping (C10, C20).

Avoidant coping strategies used by the participants included finding distractions, trying to forget the problem, dealing with problems alone, keeping quiet, leaving stressful situations, and blaming oneself. Distractions were frequently alluded to by the participants (C3, C6, C12, C17, C26, C37) and were found to be particularly helpful, for example,

We’ve been trying to teach him if you just try and do something that makes you feel good, to take your mind off it, like skateboarding. (P37)

#### The healing nature of time

Another key life lesson discussed in the interviews was the importance of the passage of time for coping with, and moving on from, stressful events. Some of the participants were acutely aware of how their feelings towards a stressor had changed over time, knowing that they felt differently now to how they felt when they had encountered a stressful situation in the past (C8), even those who had encountered very serious stressors, such as the death of a parent.

But now I deal with it so much better, it’s kind of like it happened, it’s finished, it’s done and everyone’s always like ‘are you okay’ and I’m like ‘it happened two years ago, it’s gone, don’t worry. (C1)

Mostly, parents tried to encourage their children in this behaviour as moving on was seen as advantageous, and children were aware of their parents preference for this approach (C7, C29), as opposed to dwelling on past events which ‘belong in the past’ (P32). Many parents remarked on their child’s ability to cope with stress and move on, with their children’s comments reflecting their own capacity for resilience (C2, C6, C8, C10, C13).

## Discussion

### 1. Navigating the social minefield

This theme highlighted the importance of successfully negotiating social relationships with friends, parents, and in many children’s cases, bullies.

Managing complex relationships and social hierarchies makes up a considerable proportion of the early life stress and adversity experienced by young children. This finding is supported in evolutionary psychology, with the power dynamics and social dominance created by social hierarchies regarded as a major source of psychosocial stress with health implications for childhood (Hawley, 2015) and adolescence (Koski, Xie, & Olson, 2015).

Bullying was mentioned throughout children’s narratives, which is unsurprising given that 22% of young people have reported being bullied and 22% have witnessed bullying (Annual bullying survey, 2018). It was evident that bullying had a negative psychological impact on children, principally through increasing their feelings of isolation and exclusion. Previous work suggests that if children do not learn how to cope effectively with social stressors (e.g., bullying), it can have an impact on their physiological, as well as psychological, health (Knack, Jensen‐Campbell, & Baum, 2011). Bullying and stressful social relationships were mentioned by both genders.

Interventions created to build resilience in young people often focus their efforts on increasing positive peer relationships along with other skills such as self‐efficacy, creativity, and coherence, illustrating the importance of being able to successfully navigate social relationships (Waaktaar, Christie, Helmen Borge, & Torgersen, 2004). Similar findings have been found in research with adolescents which show that close friendships and perceived friendship quality were associated with higher resilience (Graber, Turner, & Madill, 2015). Higher feelings of social belonging were also correlated with fewer instances of physical illness, showing the impact of social relationships on health (Began & Turner‐Cobb, 2012).

### 2. Pressure to thrive in the modern world

This theme demonstrated the multiple sources of pressure that children experience and how this pressure could have a positive or negative impact on children’s well‐being.

Pressure for children to thrive came from themselves and others and applied to multiple areas of their lives, including school, extracurricular activities, and taking responsibility for their own health. Research with adolescents has found similar diversity in sources of pressure, for example, peer pressure, home life, school performance, and adult responsibilities (Moksnes, Moljord, Espnes, & Byrne, 2010). These varied and substantial stressors during childhood have led some researchers to suggest that children are being ‘hurried’ to grow up too quickly (Elkind, 2001). Just as social interactions were found to be difficult to navigate, they could contribute to the pressure to achieve that children feel.

Such pressure can have a positive or negative effect on children’s self‐esteem and feelings of self‐worth, depending on their coping resources. Research has placed an emphasis on children’s personal resources for coping and resilience particularly enhancing skills such as self‐efficacy, creativity, and self‐regulation, which can help children to succeed under pressure (Lavoie, Pereira, & Talwar, 2014; Waaktaar *et al.*, 2004; Yendork & Somhlaba, 2015). The relationship between coping and self‐esteem is also bidirectional with high self‐esteem helping lead to successful coping in adolescents confronted with a social stressor (Moksnes *et al.*, 2010).

Pressure to thrive can also have a negative impact on children, leading to worry and rumination. Striving to achieve goals can be taxing on children’s coping resources (Masten, 2014). Similarly, the act of striving for perfection can cause intense distress if the goals are not achieved, with rumination acting as a mediating factor in this relationship (O’Connor, O’Connor, & Marshall, 2007).

### 3. Fear of the unknown

This theme outlined the stress created by the unknown, how children cope with familiar and novel stressors differently, with information gathering highlighted as a coping mechanism for novel stressors.

The present study suggests that not knowing the outcome of a situation can be stressful and worrying. This finding is supported in the wider literature, with fear of the unknown being a major worry during childhood hospitalization (Hart & Bossert, 1994). Coping was enhanced if the stressor was familiar rather than novel, for instance, when children encounter positive early experiences, these events shift from novel to familiar, which enhances their coping ability (Kent, Davis, & Reich, 2014). Problem‐focussed coping strategies such as information gathering are able to enhance awareness in the face of unknown stressors, for example, children awaiting surgery (Thompson, 1994).

### 4. Learning life’s lessons

This theme highlighted how children respond differently to major and minor life stresses, with learning about stress and how to cope with it given as two important life lessons, along with the healing nature of time.

Stress and coping theories have emphasized the impact of the characteristics of the stressor on coping ability, suggesting the context‐dependent nature of stress and coping (Lazarus & Folkman, 1984). The coping conceptualization used in the present research is the three‐factor model encompassing problem‐focussed coping, emotion‐focussed coping, and avoidant coping (Lazarus & Folkman, 1984; Spirito *et al.*, 1988; Turner‐Cobb & Steptoe, 1998). All three forms of coping were reported by children in the sample, with varying degrees of success in helping them cope with stress. There was no difference in the coping strategies used by boys and girls; both genders used a mixture of coping strategies. The success of different coping strategies is situational and person dependent. Successful coping could be part of the pathway to developing resilience, for example, by reducing negative chain reactions and the negative impact of stress (Rutter, 1999). Individual differences (e.g., personality), sensitivity to context and environment are also factors in the development of resilience (Boyce & Ellis, 2005; Lionetti *et al.*, 2018; Masten, 2014).

In the present study, social support from friends and family was reported as a useful coping strategy for children when faced with a variety of stressors. Much work has focussed on parental social support; however, one study linked both friend and family social support to higher levels of resilience in children who have experienced early life adversity (Yendork & Somhlaba, 2015). A review of children’s resilience and adversity found that children who relied on parental or caregiver social support coped more successfully with early life adversity (Masten, Best, & Garmezy, 1990). The involvement of parents in the development of children’s coping resources was a prominent feature in the narratives, for example, helping children to learn and use certain coping strategies such as reframing stressful experiences as positive life lessons.

The passing of time was found to help children recover from stressful experiences, perhaps by letting go of stressful experiences or developing resilience. Research has highlighted the importance of both time and stressful experiences for the development of resilience (Egeland, Carlson, & Sroufe, 1993).

### Strengths and limitations

The present study is the first to investigate children’s experiences of stress and coping using dyadic child–parent interviews, and it provides a unique discourse on issues of importance to young children. Using this method allowed for story scaffolding and a deeper understanding of child and parent perspectives on stress; these four themes formulated in analysis are supported by the wider literature. The study benefitted from a large sample size, unusual for qualitative psychology research. The sample of 38 children and 38 parents allowed for a comprehensive exploration of the topics from which complementary and divergent narratives could be established.

Despite efforts to recruit a diverse sample, participants were predominantly from higher SES backgrounds in a small relatively affluent geographical area. Future research would benefit from using a broader SES sample and sampling populations across different cultures, to examine whether the themes found in the present study apply across a broader range of backgrounds. Future research could also consider the gender of the parent being interviewed and the impact this may have on children’s narratives. Although efforts were made to reduce study fatigue, many topics were covered, in interview which could have been tiring for young children. Coverage of fewer topics may have enabled greater depth to be examined.

### Applications of the research

The findings of this research have implications for policy and practice, particularly for services that work directly with young people such as schools and mental health services (e.g., CAMHS). Considering the person and context‐dependent nature of stress and coping strategies is crucial if children are to successfully cope with and thrive following stressful early experiences. The findings from this study contribute to existing knowledge that could be used to develop a toolkit for children, their parents, schools, and services that acknowledges individual differences and parental input in coping with stress.

The use of child–parent dyadic interviews provides a valuable methodological approach in health psychology research, as evidenced by the deepened understanding of stress experience in children and the potential health interventions this enables.

## Conflict of interest

All authors declare no conflict of interest.

## COREQ criteria for reporting qualitative research

**Table:**

Domains	Item	Guide questions/description	Responses
*Domain 1: Research team and reflexivity*			
Personal characteristics	1. Interviewer/facilitator	Which author/s conducted the interview or focus group?	The lead author (TCB) conducted the interviews
	2. Credentials	What were the researcher’s credentials? For example, PhD, MD	The researcher had completed a BSc and an MSc prior to data collection
	3. Occupation	What was their occupation at the time of the study?	The researcher was a PhD student at the time of data collection
	4. Gender	Was the researcher male or female?	The researcher is female
	5. Experience and training	What experience or training did the researcher have?	The researcher had completed modules in qualitative and quantitative methods during their BSc and MSc. Further methodological training was completed during the PhD. The researcher had extensive experience working with young children and had completed previous projects with child participants
Relationship with participants	6. Relationship established	Was a relationship established prior to study commencement?	There was no relationship with participants (children and parents) prior to the study commencement
	7. Participant knowledge of the interviewer	What did the participants know about the researcher? For example, personal goals, reasons for doing the research	Before the interviews were conducted the researcher explained the study and their reasons for doing it to the participants
	8. Interviewer characteristics	What characteristics were reported about the interviewer/facilitator? For example, bias, assumptions, reasons, and interests in the research topic	The lead researcher was the interviewer in the study. Bias was reduced by using independent qualitative researchers to code a subsection of the data. No assumptions were made by the researcher. The researcher had an academic interest in the topics
*Domain 2: study design*			
Theoretical framework	9. Methodological orientation and Theory	What methodological orientation was stated to underpin the study? For example, grounded theory, discourse analysis, ethnography, phenomenology, content analysis	The study was part of an embedded multiphase design encompassing qualitative and quantitative data collection. The qualitative data were analysed using an inductive approach to thematic analysis
Participant selection	10. Sampling	How were participants selected? For example, purposive, convenience, consecutive, snowball	Participants were selected through convenience sampling
	11. Method of approach	How were participants approached? For example, face‐to‐face, telephone, mail, email	An opt‐in recruitment method using press releases in local newspapers, advertisements in schools and on relevant websites, and through social media platforms were used to recruit participants
	12. Sample size	How many participants were in the study?	Thirty‐eight children and thirty‐eight parents
	13. Non‐participation	How many people refused to participate or dropped out? Reasons?	All participants who approached the researcher about the project participated. No one withdrew from the study
Setting	14. Setting of data collection	Where was the data collected? For example, home, clinic, workplace	The interviews were carried out either in participants’ homes or in a meeting room at the university
	15. Presence of non‐participants	Was anyone else present besides the participants and researchers?	One child and one parent participant were present for each interview. In a few instances a younger sibling was present during the interview when alternative childcare arrangements could not be made. The siblings did not contribute to the interview
	16. Description of sample	What are the important characteristics of the sample? For example, demographic data, date	The participants were children aged 7–11 (16 females and 22 males) and one parent of each child (34 mothers and 4 fathers). The participants came from a high socio‐economic (SES) background. The interviews were conducted in 2015
Data collection	17. Interview guide	Were questions, prompts, guides provided by the authors? Was it pilot tested?	Interview questions and prompts were created by the researcher and can be found in Table 2 in the main article. The interview was pilot tested within the research team prior to data collection
	18. Repeat interviews	Were repeat interviews carried out? If yes, how many?	One interview per participant was carried out in the present study. Participants took part in a second brief interview in another study with the same research team (the findings of which are currently in press)
	19. Audio/visual recording	Did the research use audio or visual recording to collect the data?	Audio recording
	20. Field notes	Were field notes made during and/or after the interview or focus group?	Brief field notes were made during the interview and reflections were noted after each interview
	21. Duration	What was the duration of the interviews or focus group?	The length of the interviews ranged from 20 to 55 min depending on how much life stress children had experienced and their ability to expand on their answers, with the average length of the interviews being 30‐min
	22. Data saturation	Was data saturation discussed?	Data saturation was achieved due to the large sample size in this study
	23. Transcripts returned	Were transcripts returned to participants for comment and/or correction?	Transcripts were not returned to participants as this was not deemed appropriate for research conducted with young children
*Domain 3: analysis and findings*			
Data analysis	24. Number of data coders	How many data coders coded the data?	The lead researcher (TCB) coded all the data extracts. Two independent qualitative researchers (HF and TH) coded six of the transcripts (15% of the data) chosen at random and inter‐coder reliability was assessed
	25. Description of the coding tree	Did authors provide a description of the coding tree?	A coding manual was created which included the overarching themes, the candidate themes, definitions of each theme and examples of data extracts for each candidate theme
	26. Derivation of themes	Were themes identified in advance or derived from the data?	The themes were derived from the data as an inductive approach to thematic analysis was taken
	27. Software	What software, if applicable, was used to manage the data?	NVivo version 10
	28. Participant checking	Did participants provide feedback on the findings?	No, participants did not provide feedback on the findings
Reporting	29. Quotations presented	Were participant quotations presented to illustrate the themes/findings? Was each quotation identified? For example, participant number	Quotations are provided throughout the results of the paper to illustrate the themes. Each quotation is identified by the participant number and a ‘C’ for child participants and a ‘P’ for parents
	30. Data and findings consistent	Was there consistency between the data presented and the findings?	The themes were derived from the data therefore there is consistency between the data and findings presented. Data extracts were chosen to illustrate themes discussed in the findings
	31. Clarity of major themes	Were major themes clearly presented in the findings?	The four overarching themes are evident within the results and discussion
	32. Clarity of minor themes	Is there a description of diverse cases or discussion of minor themes?	Each overarching theme has two to four candidate themes which are clearly discussed
